# A Risk Score Model Based on Nine Differentially Methylated mRNAs for Predicting Prognosis of Patients with Clear Cell Renal Cell Carcinoma

**DOI:** 10.1155/2021/8863799

**Published:** 2021-01-14

**Authors:** Jingmin Zhou, Guanghua Liu, Xingcheng Wu, Zhien Zhou, Jialin Li, Zhigang Ji

**Affiliations:** Department of Urology, Peking Union Medical College Hospital, Chinese Academy of Medical Sciences, No. 1 Shuaifuyuan Hutong, Dongcheng District, Beijing 100730, China

## Abstract

**Purpose:**

DNA methylation alterations play important roles in initiation and progression of clear cell renal cell carcinoma (ccRCC). In this study, we attempted to identify differentially methylated mRNA signatures with prognostic value for ccRCC.

**Methods:**

The mRNA methylation and expression profiling data of 306 ccRCC tumors were downloaded from The Cancer Genome Atlas (TCGA) to screen differentially methylated lncRNAs and mRNAs (DMLs and DMMs) between bad and good prognosis patients. Uni- and multivariable Cox regression analyses and LASSO Cox-PH regression analysis were used to select prognostic lncRNAs and mRNAs. Corresponding risk scores were calculated and compared for predictive performance in the training set using Kaplan-Meier OS and ROC curve analyses. The optimal risk score was then identified and validated in the validation set. Function enrichment analysis was conducted.

**Results:**

This study screened 461 DMMs and 63 DMLs between good prognosis and bad prognosis patients, and furthermore, nine mRNAs and six lncRNAs were identified as potential prognostic molecules. Compared to nine-mRNA status risk score model, six-lncRNA methylation risk score model, and six-lncRNA status risk score model, the nine-mRNA methylation risk score model showed superiority for prognosis stratification of ccRCC patients in the training set. The prognostic ability of the nine-mRNA methylation risk score model was validated in the validation set. The nine prognostic mRNAs were functionally associated with neuroactive ligand receptor interaction and inflammation-related pathways.

**Conclusion:**

The nine-mRNA methylation signature (DMRTA2, DRGX, FAM167A, FGGY, FOXI2, KRTAP2-1, TCTEX1D1, TTBK1, and UBE2QL1) may be a useful prognostic biomarker and tool for ccRCC patients. The present results would be helpful to elucidate the possible pathogenesis of ccRCC.

## 1. Introduction

Clear cell renal cell carcinoma (ccRCC) is the most common histological subtype of renal cell carcinoma which accounts for more than 90% of cancers in the kidney [1]. ccRCC is characterized by high rates of progression and mortality, and patients have a dire prognosis [2]. Traditional clinical predictors of outcome such as clinical stages, grade, and necrosis could not achieve satisfactory results for ccRCC patients with similar clinical features but different outcomes [3]. Therefore, novel sensitive prognostic biomarkers for ccRCC are demanded.

Aberrant DNA methylation is a critical factor for initiation and progression of cancer [4]. Associations of DNA methylation with prognosis of ccRCC patients are gaining attention. For instance, based on genome-wide CpG methylation profiling, Wei et al. develop a five-CpG-based classifier as a reliable tool for survival prediction of ccRCC patients [5]. Using The Cancer Genome Atlas (TCGA) data, a recent study by Chen et al. finds that a four-gene DNA methylation signature is tightly related to prognosis of patients with kidney renal clear cell carcinoma and is able to serve as a prognostic predictor [6]. Moreover, a promoter methylation classifier panel of 172 differentially methylated CpGs is reported to be capable of classifying prognosis of nonmetastatic ccRCC patients [7]. Long noncoding RNAs (lncRNAs) modulate gene expression at various levels and facilitate cancer phenotypes via interaction with other cellular macromolecules, such as DNA, RNA, and protein [8, 9]. Important implications of lncRNAs have been demonstrated for tumorigenesis of ccRCC [10]. A large number of lncRNAs gain DNA methylation in ccRCC, and several lncRNAs with promoter methylation are related to outcome of patients [11]. However, aberrantly methylated lncRNAs which can function as prognostic biomarkers for ccRCC patients remain elusive.

In this paper, we tried to identify potential mRNA methylation signature and lncRNA methylation signature for survival prediction of ccRCC patients through a comprehensive analysis of mRNA and lncRNA methylation data downloaded from TCGA dataset, and the predictive performances of methylation or status risk scores were compared based on these prognostic lncRNAs or mRNAs to select the best prognostic model for ccRCC.

## 2. Materials and Methods

### 2.1. Datasets and Preprocessing

Methylation and gene expression data of 309 ccRCC tumor tissue samples and 24 normal control tissue samples were downloaded from TCGA repository using Illumina Infinium Human Methylation 450 BeadChip platform and Illumina HiSeq 2000 RNA Sequencing platform. Patients without complete survival information were excluded from the study, and the resulting 306 patients with ccRCC tumor tissue samples were used as the training set in the current study. E-MTAB-3274 [12] dataset consisting of methylome data of 107 ccRCC samples was obtained from EBI ArrayExpress (https://www.ebi.ac.uk/arrayexpress/) based on Illumina Infinium Human Methylation 450 BeadChip platform. Of these samples, 102 samples had corresponding survival information and were used as the validation set.

According to probe sites and ID information, we identified lncRNAs and mRNAs from TCGA set and E-MTAB-3274 dataset based on HUGO Gene Nomenclature Committee (HGNC) [13] database (http://www.genenames.org/) that enrolls 4112 lncRNAs and 19201 protein coding genes. As a result, 13919 mRNAs and 1028 lncRNAs were identified.

### 2.2. Differential Methylation Analysis between Good and Bad Prognosis Samples

In the training set, differentially methylated lncRNAs and mRNAs (DMLs and DMMs) were identified between good prognosis patients (being alive with OS time lasting for more than 60 months) and bad prognosis patients (being dead with OS time lasting for less than 36 months). Significant DMLs and DMMs were defined as those with FDR < 0.05 and ∣log_2_FC | >0.263. Next, two-way hierarchical clustering analysis of these significant DMLs and DMMs was carried out based on centered Pearson correlation algorithm.

### 2.3. Correlation Analysis between DNA Methylation and Expression of Significant DMLs and DMMs

We analyzed relationships of overall methylation and expression levels of preselected significant DMLs and DMMs through calculating Pearson correlation coefficient (PCC) and Spearman correlation coefficient (SCC) [14]. Subsequently, PCC of methylation and expression levels of each individual DML or DMM was computed. These DMLs and DMMs with significant negative correlations (*p* value <0.05) were selected to be applied in further analysis.

### 2.4. Prognostic Risk Scoring Model Building

In order to construct risk scoring models for predicting survival in ccRCC patients, we employed a step-by-step strategy to identify the prognostic DMLs and DMMs in the training set. Firstly, we performed univariable Cox regression analysis to assess the association between methylation levels of the aforementioned DMLs and DMMs with patients' OS time. The resulting lncRNAs and mRNAs were considered significant if their *p* values <0.05. Secondly, the significant lncRNAs and mRNAs were chosen to undergo a multivariable Cox regression analysis. The significant RNAs (*p* value <0.05) were determined to be independent predictors of prognosis and were then used to fit a L1-penalized (LASSO) Cox-PH regression [15] model with estimation of optimal lambda value through performing 1,000 cross-validations. The optimal feature combination was selected using LASSO regularized regression algorithm in penalized package. CV and lambda values were obtained by feature filtering. The small lambda value usually resulted in small penalty term value as well as over fitting phenomenon; therefore, we chose the largest lambda value which is corresponding to the lambda parameter value. Eventually, the resulting optimal set of predictive lncRNAs and mRNAs was applied to build risk scoring models based on their methylation levels or status together with the estimated multivariable Cox regression coefficients. Methylation status of an individual lncRNA or mRNA was defined according to optimal cutoff point of methylation value (Monte-Carlo *p* value <0.05) which was determined based on patients' survival through performing X-Tile Bio-Informatics Tool [16] (high expression, expression status = 1; low expression, expression status = 0). We utilized the following equations to quantify methylation or status risk scores for survival prediction:

Status risk score = ∑*β* RNAn × status RNAn,

Methylation risk score = ∑(*β* RNAn × methylation RNAn).

*β* RNAn suggests regression coefficient of RNAn; status RNAn and methylation RNAn suggest methylation status and values of RNAn, respectively.

Risk score was estimated for each patient using above formula. With median risk score as cutoff point, the training set or the validation set was dichotomized into a high-risk group and a low-risk group.

### 2.5. Statistical Analysis

Kaplan-Meier survival plots as well as log-rank test were used to analyze difference in OS time between two risk groups. Receiver operating characteristic (ROC) curve was plotted to compare sensitivity and specificity of different risk scores in predicting OS time of patients. Based on the training set, associations of clinical features and risk score status with patients' OS were calculated using uni- and multivariable Cox regression analyses. Nomogram was built combining the optimal risk score status with prognostic clinical features. Calibration plots were used to evaluate predictive capability of the nomogram. Different packages of the R software (version 3.4.1) were used to implement all these bioinformatics and statistical analyses in the study: limma [17] package for differential methylation analysis and differential expression analysis; pheatmap [18] package (https://cran.r-project.org/ web/packages/pheatmap/index.html) for hierarchical clustering analysis; survival package (version 2.41-1, http://bioconductor.org/packages/survivalr/) for uni- and multivariable Cox regression analyses and Kaplan-Meier OS analysis; cor.test (https://stat.ethz.ch/R-manual/R-devel/library/stats/html/cor. test. html) function for calculation of PCC and SCC values; pROC package for ROC curve analysis; penalized package (version 0.9.50) for LASSO Cox-PH regression model; rms package [19] (version 5.1-2, https://cran.r-project.org/web/packages/rms/index.html) for nomogram analysis; gene set enrichment analysis (GSEA) [20] software (http://software.broadinstitute.org/gsea/index.jsp) for Kyoto Encyclopedia of Genes and Genomes (KEGG) pathway enrichment analysis. Significance was defined as *p* value <0.05.

### 2.6. Functional Enrichment Analysis

According to the optimal risk score, the training set was divided into a high-risk group and a low-risk group. Between the two risk groups, differentially expressed genes (DEGs) were identified based on the threshold of FDR < 0.05 and ∣log_2_FC | >0.5 and then underwent KEGG pathway enrichment analysis. Significant signaling pathways were selected in the present study.

## 3. Results

### 3.1. Detecting DMLs and DMMs between Good Prognosis and Bad Prognosis Patients

**Figure 1:**
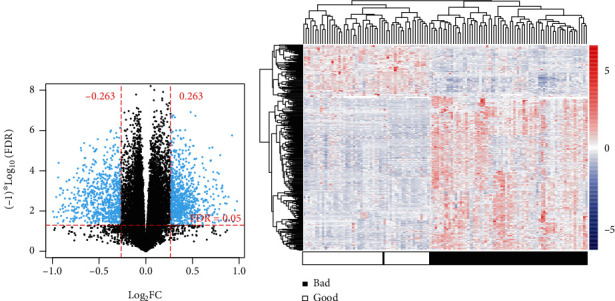
Graphical display of differentially methylated RNAs between good prognosis and bad prognosis samples. (a) Volcano plot of effect size (log_2_FC) − log_10_(FDR) of the identified differentially methylated RNAs. (b) Two-way hierarchical clustering heatmap for the identified differentially methylated RNAs.

The training set was consisted of 68 bad prognosis patients and 52 good prognosis patients. As shown in Figures 1(a) and 1(b), we found a total of 524 DMRs (FDR < 0.05 and ∣log_2_FC | >0.263) between the good prognosis patients and the bad prognosis patients, including 461 DMMs and 63 DMLs. Furthermore, the 461 DMMs were composed of 119 hypomethylated mRNAs and 342 hypermethylated mRNAs, and the 63 DMLs were comprised of 34 hypomethylated lncRNAs and 29 hypermethylated lncRNAs. Results of two-way hierarchical clustering analysis for these patients were shown in a heatmap (Figure 1(b)). Based on methylation value, ccRCC patients were categorized into two groups.

### 3.2. Correlation between DNA Expression and Methylation

For the total of 524 identified DMRs, the overall methylation level was negatively correlated with the overall expression level (PCC = −0.1909, *p* value = 1.252*E*^−04^; SCC = −0.1456, *p* value = 3.589*E*^−04^). A significant negative correlation between methylation and expression of each individual DMR was observed for 222 DMRs, consisting of 27 lncRNAs and 195 mRNAs. These 222 DMRs were selected for further analysis.

### 3.3. Identifying a Nine-mRNA Methylation Signature and a Six-lncRNA Methylation Signature for Survival Prediction

Using data in the training set, we performed univariable Cox regression analysis of the 222 DMRs. Totally 124 resulting DMRs were significantly correlated with patients' OS time (*p* value <0.05), including 113 mRNAs and 11 lncRNAs. Furthermore, 31 DMRs were determined to be independent prognostic factors in multivariable Cox regression analysis (*p* value <0.05), consisting of 24 mRNAs and 7 lncRNAs. Consequently, the 31 DMRs were used to fit a LASSO Cox-PH regression model. Using the optimal lamda value, as shown in Table 1 and supplement Figure 1 (Figure S1), we retrieved an optimal panel of 9 predictive mRNAs (lamda = 0.51184, CVL = −546.8306; DMRTA2, DRGX, FAM167A, FGGY, FOXI2, KRTAP2-1, TCTEX1D1, TTBK1, and UBE2QL) and an optimal panel of 6 predictive lncRNAs (lamda = 0.03557, CVL = −557.1598; FAM138B, HCG11, KIAA0087, MIAT, PSORS1C3, and SNHG11).

Based on methylation levels or status of the 9 prognostic mRNAs or the 6 prognostic lncRNAs and their multivariable Cox regression coefficients, we designed formulas for calculating methylation or status risk scores as follows:

mRNA status risk score = (0.447514)∗status_DMRTA2_ + (−1.145573)∗status_DRGX_ + (−0.280914)∗status_FAM167A_ + (1.593956)∗status_FGGY_ + (0.034273)∗status_FOXI2_ + (0.161233)∗status_KRTAP2−1_ + (0.524107)∗status_TCTEX1D1_ + (0.43882)∗status_TTBK1_ + (0.577028)∗status_UBE2QL1_,

lncRNA status risk score = (1.349587)∗status_FAM138B_ + (0.278684)∗status_HCG11_ + (−2.97586)∗status_KIAA0087_ + (1.262154)∗status_MIAT_ + (−1.687644)∗status_PSORS1C3_ + (−1.199576)∗status_SNHG11_,

mRNA methylation risk score = (0.447514)∗methy_DMRTA2_ + (−1.145573)∗methy_DRGX_ + (−0.280914)∗methy_FAM167A_ + (1.593956)∗methy_FGGY_ + (0.034273)∗methy_FOXI2_ + (0.161233)∗methy_KRTAP2−1_ + (0.524107)∗methy_TCTEX1D1_ + (0.43882)∗methy_TTBK1_ + (0.577028)∗methy_UBE2QL1_,

lncRNA methylation risk score = (1.349587)∗methy_FAM138B_ + (0.278684)∗methy_HCG11_ + (−2.97586)∗methy_KIAA0087_ + (1.262154)∗methy_MIAT_ + (−1.687644)∗methy_PSORS1C3_ + (−1.199576)∗methy_SNHG11_.

**Figure 2:**
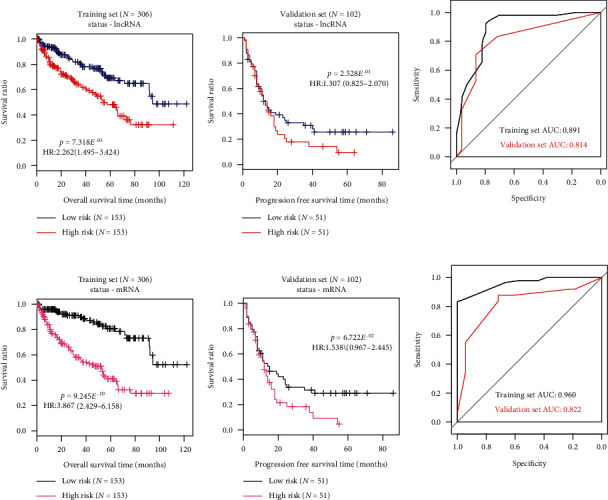
Kaplan-Meier survival and ROC curves for (a) six-lncRNA status risk score model and (b) nine-mRNA status risk score model in the training set and the validation set.

**Figure 3:**
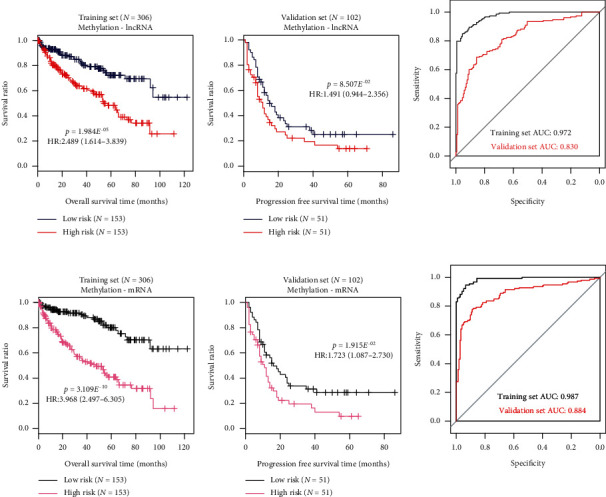
Kaplan-Meier survival and ROC curves for six-lncRNA methylation risk score model and nine-mRNA methylation risk score model in the training set and the validation set. (a) Six-lncRNA status risk score; (b) nine-mRNA status risk score.

Nine-mRNA methylation risk score, nine-mRNA status risk score, six-lncRNA methylation risk score, and six-lncRNA status risk score were calculated for each patient in the training set, respectively. With median risk score as threshold, the training set was divided into a high-risk group and a low-risk group. For each risk score, difference in OS time was significant between the two risk groups according to results of Kaplan-Meier survival analysis and log-rank test (Figures 2 and 3). The nine-mRNA methylation risk score displayed markedly smaller *p* value than the other three risk scores (nine-mRNA methylation risk score, HR (95%CI) = 3.968 (2.497 − 6.305) and *p* value = 3.109*E*^−10^; six-lncRNA methylation risk score, HR (95%CI) = 2.489 (1.614 − 3.839) and *p* value = 1.984*E*^−05^; nine-mRNA status risk score, HR (95%CI) = 3.867 (2.429 − 6.158) and *p* value = 9.245*E*^−10^; six-lncRNA status risk score, HR (95%CI) = 2.262 (1.495 − 3.424) and *p* value = 7.318*E*^−05^, Table 2). Validation was performed in the validation set to assess predictive performance of the four risk scores. Only the nine-mRNA methylation risk score could dichotomize the validation set into two risk groups with significantly different OS time (nine-mRNA methylation risk score, HR(95%CI) = 1.723 (1.087 − 2.730) and *p* value = 1.915*E*^−02^; six-lncRNA methylation risk score, HR(95%CI) = 1.491 (0.944 − 2.356) and *p* value = 8.507*E*^−02^; nine-mRNA status risk score, HR(95%CI) = 1.538 (0.967 − 2.445) and *p* value = 6.722*E*^−02^; six-lncRNA status risk score, HR(95%CI) = 1.307 (0.825 − 2.070) and *p* value = 2.528*E*^−01^, Table 2).

Results of ROC curve analysis were depicted in Figures 2 and 3 and Table 3. AUC values of nine-mRNA methylation risk score for the training set and the validation set were 0.987 and 0.884, respectively, which are higher compared to the other risk scores. All above observations suggest that the nine-mRNA methylation risk score performs better than other risk scores for survival prediction of ccRCC patients.

### 3.4. Characterization of Four Independent Prognostic Features and Construction of a Composite Nomogram

**Figure 4:**
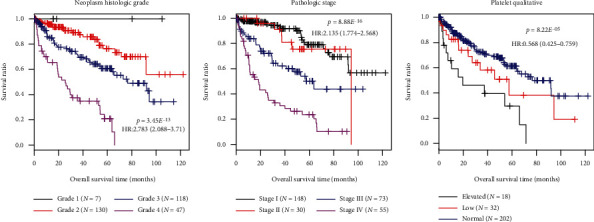
Kaplan-Meier survival curves for the training set classified by neoplasm histologic grade, pathologic stage, and platelet qualitative. (a) Neoplasm histologic grade; (b) pathologic stage; (c) platelet qualitative. Patients of the training set are separated by neoplasm histologic grade, pathologic stage, or platelet qualitative into different subgroups, separately. OS time of different subgroups is compared and analyzed using Kaplan-Meier survival analysis.

**Figure 5:**
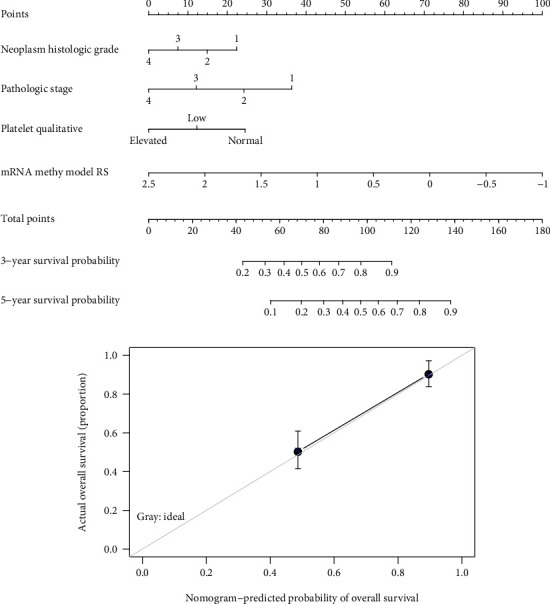
Nomogram for predicting survival of ccRCC patients. (a) The nomogram combines nine-mRNA methylation model, neoplasm histologic grade, pathologic stage, and platelet qualitative. (b) Calibration plots for predicting 5-year OS. Grey line, ideal agreement between the actual and predicted probabilities of 5-year OS; solid line, the actual nomogram.

The univariable Cox regression analysis in the training set indicated that age, histologic grade, pathologic N, pathologic T, pathologic stage, hemoglobin, platelet qualitative, and nine-mRNA methylation risk score were significantly related to patients' OS time (*p* value <0.05, Table 4). Furthermore, histologic grade (HR = 1.470, 95%CI = 1.076 − 2.009, and *p* value = 1.56*E*^−02^), pathologic stage (HR = 1.989, 95%CI = 1.101 − 3.592, and *p* value = 2.26*E*^−02^), platelet qualitative (HR = 0.690, 95%CI = 0.501 − 0.949, and *p* value = 2.28*E*^−02^), and nine-mRNA methylation risk score model (HR = 2.574, 95%CI = 1.149 − 5.767, and *p* value = 2.17*E*^−04^) were found to be independent predictive factors of prognosis in multivariable Cox regression analysis (Table 4, Figure 4). It revealed that prognostic value of the nine-mRNA methylation risk score model was independent of clinical factors. To integrate the nine-mRNA methylation risk score with the three prognostic clinical variables, a composite nomogram was built (Figure 5(a)). Calibration curves for 5-year survival probability showed sound agreement between predicted and actual probabilities of 5-year survival (Figure 5(b)).

### 3.5. Functional Implications of the Nine Prognostic mRNAs

**Figure 6:**
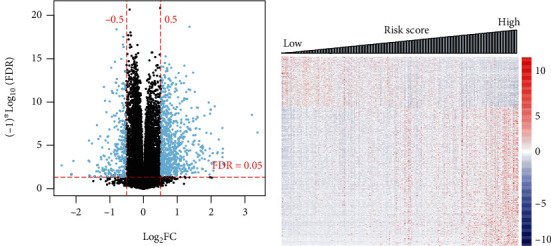
Identification of DEGs between the high-risk and low-risk groups. (a) Volcano plot of effect size (log_2_FC) − log_10_(FDR) of the DEGs. (b) Risk score distribution of these DEGs expression profiles.

To investigate functional characteristics of the nine prognostic mRNAs in ccRCC biology, we performed functional enrichment analysis on the identified DEGs between the high-risk group and the low-risk group of the training set classified by the nine-mRNA methylation risk score. We found 715 DEGs between the two risk groups, consisting of 190 downregulated genes and 525 upregulated genes (Figures 6(a) and 6(b)). These genes were significantly involved in cytokine-cytokine receptor interaction, neuroactive ligand receptor interaction, toll-like receptor signaling pathway, and cell receptor signaling pathway (Table 5).

## 4. Discussion

Considering the prognostic potential of DNA methylation for ccRCC, the present work conducted a genome-wide analysis of mRNAs and lncRNAs methylation profiling of ccRCC samples from TCGA systematically, with a view to discover novel and reliable prognostic biomarkers. There were 461 DMMs and 63 DMLs between good prognosis and bad prognosis patients. Among which, 24 DMMs and 7 DMLs were identified to be independent predictive factors by uni- and multivariable Cox regression analyses. Eventually, a nine-mRNA methylation signature and a six-lncRNA methylation signature were determined by LASSO Cox-PH regression model. Four prognostic prediction systems were established, and four risk score models were generated, including nine-mRNA methylation risk score model, nine-mRNA status risk score model, six-lncRNA methylation risk score model, and six-lncRNA status risk score model. The nine-mRNA methylation risk score model performed better than other DML-based risk scores in predicting survival outcome of ccRCC patients. This study suggests that the nine-mRNA methylation risk score is reliable and powerful for classifying ccRCC patients according to prognosis.

Our study identified a prognostic nine-mRNA methylation signature, comprising of DMRTA2, DRGX, FAM167A, FGGY, FOXI2, KRTAP2-1, TCTEX1D1, TTBK1, and UBE2QL1. DMRTA2, a member of the DMRT family, is involved in central nervous system development [21]. Methylation of DMRTA2 is associated with survival of patients with triple-negative breast cancer [22]. DRGX encodes dorsal root ganglia homeobox protein that is a transcription factor implicated in embryonic development of the nervous system. There is evidence that DGRX may be related to serum concentration of monocyte chemoattractant protein-1, an inflammation biomarker, in subjects treated with fenofibrate, an anti-inflammatory and triglyceride-lowering drug [23]. FAM167A, also namely C8orf13, belongs to the FAM167 (SEC) family. The polymorphisms of FAM167A-BLK region are linked to susceptibility of autoimmune diseases [24, 25]. Low expression of FAM167A is related to longer survival time of ccRCC patients [26]. FGGY encodes protein FGGY carbohydrate kinase domain containing proteins that phosphorylate carbohydrates [27]. FGGY is significantly upregulated during neurogenic skeletal muscle atrophy [28]. FOX family members play roles in regulating cell growth and differentiation [29], and FOXI2 expression is associated with survival of ccRCC patients [30]. KRTAP proteins are major components of hair shaft, playing crucial roles in human hair shaft keratinization [31]. KRTAP2-1 shows an association with chemosensitivity of colorectal cells to oxaliplatin [32]. TCTEX1D family members contain a conserved domain similar to C-terminus of TCTEX1, with little information regarding their biological function [33]. TCTEX1D1 is found to be hypermethylated and downregulated in endometrial cancer [34]. Tau Tubulin Kinase 1 encoded by TTBK1 is implicated in regulating microtube assembly and stabilization. TTBK1 phosphorylation activity is linked to several neurodegenerative diseases like Alzheimer's disease [35]. UBE2QL1 protein is related to protein ubiquitination. There is evidence that UBE2QL1 is a candidate renal tumor suppressor gene [36]. Aberrant methylation of UBE2QL1 has been found in HBV-related hepatocellular carcinoma [37]. As far as we know, for the first time, the nine differentially methylated mRNAs are reported to be of prognostic value for ccRCC patients.

The training set of this study was dichotomized by the nine-mRNA methylation risk score model into a high-risk group and a low-risk group. Function enrichment analysis showed that the DEGs between the two risk groups were significantly enriched in cytokine-cytokine receptor interaction, neuroactive ligand receptor interaction, toll-like receptor signaling pathway, and cell receptor signaling pathways. Mounting evidences demonstrate that neuroactive ligand receptor interaction pathway is an important pathway for ccRCC [38, 39]. Toll-like receptors are critical participants in inflammation responses. Cancer-related inflammation has been accepted as an important hallmark of cancer [40]. These findings reveal that the nine prognostic mRNAs may be involved in neuroactive ligand receptor interaction and inflammation-related pathways. Further functional annotation of these mRNAs is needed to deepen our understanding of their biological implications in ccRCC prognosis.

It should be noted that this study is an extensive bioinformatics study based on published data. The results of these studies should also be further validated *in vitro* or *in vivo* models. We hope that these useful clues will help other researchers to carry out relevant research.

## 5. Conclusion

We report and validate predictive value of a nine-mRNA methylation risk score model for risk stratification of ccRCC patients. The nine-mRNA methylation signature, including DMRTA2, DRGX, FAM167A, FGGY, FOXI2, KRTAP2-1, TCTEX1D1, TTBK1, and UBE2QL1, may be a useful prognostic biomarker for ccRCC patients. Further experimental investigations are required to confirm this prognostic signature.

## Figures and Tables

**Table 1:** Information of the six-lncRNA signature and the nine-mRNA signature.

Type	ID	Locus	Coefficient	HR	95% CI	Value	Cutoff
lncRNA	FAM138B	cg08267300	1.3497	4.436	1.388-6.461	1.31 × 10^−3^	0.6
	HCG11	cg27490387	0.2787	2.181	1.158-4.108	2.20 × 10^−2^	0.10
	KIAA0087	cg25962015	-2.9759	0.043	0.003-0.532	7.10 × 10^−3^	0.72
	MIAT	cg23345500	1.2621	1.408	1.064-3.084	4.66 × 10^−2^	0.04
	PSORS1C3	cg26504835	-1.6876	0.047	0.006-0.350	1.41 × 10^−3^	0.32
	SNHG11	cg27314048	-1.1996	0.111	0.013-0.988	2.47 × 10^−2^	0.85

mRNA	DMRTA2	cg27265170	0.4475	3.839	1.078-4.366	4.54 × 10^−2^	0.23
	DRGX	cg17494438	-1.1456	0.038	0.020-0.718	2.91 × 10^−2^	0.47
	FAM167A	cg02771117	-0.2809	8.842	3.902-10.04	1.42 × 10^−2^	0.41
	FGGY	cg27571329	1.5940	4.524	3.678-5.565	6.08 × 10^−4^	0.69
	FOXI2	cg26115633	0.0342	2.546	1.086-5.971	4.43 × 10^−2^	0.55
	KRTAP2-1	cg24624629	0.1612	1.385	1.195-5.194	7.66 × 10^−3^	0.35
	TCTEX1D1	cg25339566	0.5241	2.711	1.670-4.396	2.03 × 10^−2^	0.10
	TTBK1	cg27363327	0.4388	0.152	0.084-0.275	4.60 × 10^−3^	0.12
	UBE2QL1	cg26692294	0.5770	0.063	0.012-0.342	1.29 × 10^−2^	0.37

HR: hazard ratio; CI: confidence interval.

**Table 2:** Results of Kaplan-Meier OS analysis for four risk scores.

Type	Training set		Validation set	
	*p* value	HR (95% CI)	*p* value	HR (95% CI)
Six-lncRNA status risk score	7.318 × 10^−5^	2.262 (1.495-3.424)	2.528 × 10^−1^	1.307 (0.825-2.070)
Nine-mRNA status risk score	9.245 × 10^−10^	3.867 (2.429-6.158)	6.722 × 10^−2^	1.538 (0.967-2.445)
Six-lncRNA methylation risk score	1.984 × 10^−5^	2.489 (1.614-3.839)	8.507 × 10^−2^	1.491 (0.944-2.356)
Nine-mRNA methylation risk score	3.109 × 10^−10^	3.968 (2.497-6.305)	1.915 × 10^−2^	1.723 (1.087-2.730)

HR: hazard ratio; CI: confidence interval.

**Table 3:** AUC values of ROC curves for four risk scores.

Type	Training set	Validation set
Six-lncRNA status risk score	0.891	0.814
Nine-mRNA status risk score	0.960	0.822
Six-lncRNA methylation risk score	0.972	0.830
Nine-mRNA methylation risk score	0.987	0.884

**Table 4:** Uni- and multivariable Cox regression analyses in the training set.

Clinical characteristics	Training set (*N* = 306)	Univariable Cox			Multivariable Cox		
		HR	95% CI	*p* value	HR	95% CI	*p* value
Age (years, mean ± SD)	64.57 ± 11.79	1.777	1.171-2.697	6.21 × 10^−3^	1.352	0.846-2.158	2.07 × 10^−1^
Gender (male/female)	200/106	1.141	0.746-1.743	5.43 × 10^−1^	-	-	-
Neoplasm histologic grade (G1/G2/G3/G4/-)	7/130/118/47/4	2.783	2.088-3.710	3.45 × 10^−13^	1.470	1.076-2.009	1.56 × 10^−2^
Pathologic M (M0/M1/-)	229/52/25	4.720	3.115-7.153	7.77 × 10^−16^	1.034	0.403- 2.657	1.36 × 10^−1^
Pathologic N (N0/N1/-)	130/9/167	2.617	1.030-6.651	3.57 × 10^−2^	0.462	0.0985-2.171	3.28 × 10^−1^
Pathologic T (T1/T2/T3/T4)	151/40/107/8	2.101	1.678-2.630	5.38 × 10^−12^	0.753	0.456-1.243	2.65 × 10^−1^
Pathologic stage (I/II/III/IV)	148/30/73/55	2.135	1.774-2.568	8.88 × 10^−16^	1.989	1.101-3.592	2.26 × 10^−2^
Hemoglobin (elevated/low/normal/-)	5/151/104/46	0.423	0.276-0.646	5.54 × 10^−5^	0.466	0.269-1.087	3.86 × 10^−1^
Platelet qualitative (elevated/low/normal/-)	18/32/202/54	0.568	0.425-0.759	8.22 × 10^−5^	0.690	0.501- 0.949	2.28 × 10^−2^
White cell count (elevated/low/normal/-)	84/4/161/57	1.156	0.910-1.468	2.33 × 10^−1^	-	-	-
Serum calcium (elevated/low/normal/-)	5/112/90/99	1.017	0.658-1.570	9.40 × 10^−1^	-	-	-
Methylation RS model (high/low)	153/153	4.713	2.898-.665	6.50 × 10^−12^	2.574	1.149-5.767	2.17 × 10^−4^
Vital status (dead/alive)	97/209	-	-	-	-	-	-
Overall survival time (months, mean ± SD)	35.21 ± 28.73	-	-	-	-	-	-

**Table 5:** Significant enriched KEGG pathways.

Name	Size	ES	NES	NOM *p* value	Enriched genes
KEGG cytokine-cytokine receptor interaction	18	0.5690	1.9390	0	IL6, IL1R2, IL2RA, CCL13, IL20RB, CXCL13, CXCR3, IL21R, TNFRSF17, TNFSF14, TNFRSF9, TNFRSF18, INHBE, IFNG, XCL2, LTA, CD27, XCL1
KEGG neuroactive ligand receptor interaction	11	-0.4944	-1.7734	0.0108	GABRB3, CHRM3, VIPR1, GRIK3, PLG, CALCR, GABRQ, AVPR2, TACR1, AGTR1, F2RL3
KEGG toll-like receptor signaling pathway	2	0.8721	1.5058	0.0188	LBP, IL6
KEGG B cell receptor signaling pathway	3	0.7853	1.5119	0.0448	CD79A, CD72, CARD11

ES: enrichment score; NES: normalized enrichment score; NOM *p* value: nominal *p* value; size: the number of genes enriched in one pathway.

## Data Availability

All data used and/or analyzed in this study are available from TCGA database (https://portal.gdc.cancer.gov) or the EBI Array database (https://www.ebi.ac.uk/arrayexpress/).

## Supplementary Materials

Supplementary Materials Figure S1: the lambda parameter curve (left) and coefficient distribution chart (right) of (a) lncRNA and (b) mRNA. The lambda parameter curve was gained basing on cross-validation likelihood screening. The horizontal axis and vertical axis represent the different values of lambda and CVL, respectively, and the intersection of red dotted lines indicates the value of lambda parameters when CVL reaches the maximum value. The coefficient distribution charts for optimal prognosis were screened basing on Cox-PH model using L1-penalized regularized regression algorithm.

## References

[1] Hsieh J. J., Purdue M. P., Signoretti S. (2017). Renal cell carcinoma. *Nature reviews Disease primers*.

[2] Ljungberg B., Bensalah K., Canfield S. (2015). EAU guidelines on renal cell carcinoma: 2014 update. *European Urology*.

[3] Zigeuner R., Hutterer G., Chromecki T. (2010). External validation of the Mayo Clinic stage, size, grade, and necrosis (SSIGN) score for clear-cell renal cell carcinoma in a single European centre applying routine pathology. *European Urology*.

[4] Klutstein M., Nejman D., Greenfield R., Cedar H. (2016). DNA methylation in cancer and aging. *Cancer Research*.

[5] Wei J. H., Haddad A., Wu K. J. (2015). A CpG-methylation-based assay to predict survival in clear cell renal cell carcinoma. *Nature Communications*.

[6] Chen G., Wang Y., Wang L., Xu W. (2017). Identifying prognostic biomarkers based on aberrant DNA methylation in kidney renal clear cell carcinoma. *Oncotarget*.

[7] Evelönn E. A., Landfors M., Haider Z. (2019). DNA methylation associates with survival in non-metastatic clear cell renal cell carcinoma. *BMC Cancer*.

[8] Cheetham S., Gruhl F., Mattick J., Dinger M. (2013). Long noncoding RNAs and the genetics of cancer. *British Journal of Cancer*.

[9] Kim E.-D., Sung S. (2012). Long noncoding RNA: unveiling hidden layer of gene regulatory networks. *Trends in Plant Science*.

[10] Liu X., Hao Y., Yu W. (2018). Long non-coding RNA emergence during renal cell carcinoma tumorigenesis. *Cellular Physiology and Biochemistry : International Journal of Experimental Cellular Physiology, Biochemistry, and Pharmacology*.

[11] Malouf G., Zhang J., Tannir N. M. (2015). Charting DNA methylation of long non-coding RNA in clear-cell renal cell carcinoma. *Journal of Clinical Oncology*.

[12] Beuselinck B., Job S., Becht E. (2015). Molecular subtypes of clear cell renal cell carcinoma are associated with sunitinib response in the metastatic setting. *Clinical Cancer Research : an official journal of the American Association for Cancer Research*.

[13] Wright M. W. (2014). A short guide to long non-coding RNA gene nomenclature. *Human Genomics*.

[14] Zou K. H., Tuncali K., Silverman S. G. (2003). Correlation and simple linear regression. *Radiology*.

[15] Huang S., Yee C., Ching T., Yu H., Garmire L. X. (2014). A novel model to combine clinical and pathway-based transcriptomic information for the prognosis prediction of breast cancer. *PLoS Computational Biology*.

[16] Pan C., Wang X., Chen W. (2015). Reevaluation of glypican-3 as a prognostic marker in HCC using X-tile software. *Medical Oncology*.

[17] Ritchie M. E., Phipson B., Di Wu Y. H., Law C. W., Shi W., Smyth G. K. (2015). limma powers differential expression analyses for RNA-sequencing and microarray studies. *Nucleic Acids Research*.

[18] Yang L., Xu L., Wang Q., Wang M., An G. (2016). Dysregulation of long non-coding RNA profiles in human colorectal cancer and its association with overall survival. *Oncology Letters*.

[19] Zhang Z., Kattan M. W. (2017). Drawing nomograms with R: applications to categorical outcome and survival data. *Annals of Translational Medicine*.

[20] Subramanian A., Tamayo P., Mootha V. K. (2005). Gene set enrichment analysis: a knowledge-based approach for interpreting genome-wide expression profiles. *Proceedings of the National Academy of Sciences*.

[21] Poulain M., Frydman N., Tourpin S. (2014). Involvement of doublesex and mab-3-related transcription factors in human female germ cell development demonstrated by xenograft and interference RNA strategies. *Molecular Human Reproduction*.

[22] Stirzaker C., Zotenko E., Song J. Z. (2015). Methylome sequencing in triple-negative breast cancer reveals distinct methylation clusters with prognostic value. *Nature Communications*.

[23] Aslibekyan S., Kabagambe E. K., Irvin M. R. (2012). A genome-wide association study of inflammatory biomarker changes in response to fenofibrate treatment in the Genetics of Lipid Lowering Drug and Diet Network. *Pharmacogenetics and Genomics*.

[24] Song R.-h., Li Q., Jia X., Yao Q.-m., Wang B., Zhang J.-a. (2018). Polymorphisms of FAM167A-BLK region confer risk of autoimmune thyroid disease. *DNA and Cell Biology*.

[25] Chen S., Wu W., Li J. (2015). Single nucleotide polymorphisms in the FAM167A-BLK gene are associated with polymyositis/dermatomyositis in the Han Chinese population. *Immunologic Research*.

[26] Wang Y., Wang Y., Liu F. (2018). A 44-gene set constructed for predicting the prognosis of clear cell renal cell carcinoma. *International Journal of Molecular Medicine*.

[27] Zhang Y., Zagnitko O., Rodionova I., Osterman A., Godzik A. (2011). The FGGY carbohydrate kinase family: insights into the evolution of functional specificities. *PLoS Computational Biology*.

[28] De Las Casas B., Waddell D. (2016). FGGY carbohydrate kinase domain containing is upregulated during neurogenic skeletal muscle atrophy. *The FASEB Journal*.

[29] Katoh M., Igarashi M., Fukuda H., Nakagama H., Katoh M. (2013). Cancer genetics and genomics of human FOX family genes. *Cancer Letters*.

[30] Jia Z., Wan F., Zhu Y. (2018). Forkhead-box series expression network is associated with outcome of clear-cell renal cell carcinoma. *Oncology Letters*.

[31] Fujikawa H., Fujimoto A., Farooq M., Ito M., Shimomura Y. (2012). Characterization of the human hair keratin–associated protein 2 (KRTAP2) gene family. *Journal of Investigative Dermatology*.

[32] Zhang Y.-J., Li A.-J., Han Y., Yin L., Lin M.-B. (2014). Inhibition of Girdin enhances chemosensitivity of colorectal cancer cells to oxaliplatin. *World Journal of Gastroenterology*.

[33] Gholkar A. A., Senese S., Lo Y.-C. (2015). Tctex1d2 associates with short-rib polydactyly syndrome proteins and is required for ciliogenesis. *Cell Cycle*.

[34] Men C., Chai H., Song X., Li Y., Du H., Ren Q. (2017). Identification of DNA methylation associated gene signatures in endometrial cancer via integrated analysis of DNA methylation and gene expression systematically. *Journal of Gynecologic Oncology*.

[35] Nozal V., Martinez A. (2018). Tau Tubulin Kinase 1 (TTBK1), a new player in the fight against neurodegenerative diseases. *European Journal of Medicinal Chemistry*.

[36] Wake N. C., Ricketts C. J., Morris M. R. (2013). UBE 2 QL 1 is disrupted by a constitutional translocation associated with renal tumor predisposition and is a novel candidate renal tumor suppressor gene. *Human Mutation*.

[37] Ye C., Tao R., Cao Q. (2016). Whole-genome DNA methylation and hydroxymethylation profiling for HBV-related hepatocellular carcinoma. *International Journal of Oncology*.

[38] Liu X., Wang J., Sun G. (2015). Identification of key genes and pathways in renal cell carcinoma through expression profiling data. *Kidney and Blood Pressure Research*.

[39] Yang W., Yoshigoe K., Qin X. (2014). Identification of genes and pathways involved in kidney renal clear cell carcinoma. *BMC Bioinformatics*.

[40] Chang Y., An H., Xu L. (2015). Systemic inflammation score predicts postoperative prognosis of patients with clear-cell renal cell carcinoma. *British Journal of Cancer*.

