# Supplementary figures and images for: A Risk Score Model Based on Nine Differentially Methylated mRNAs for Predicting Prognosis of Patients with Clear Cell Renal Cell Carcinoma
Source: Dis Markers. 2021 Jan 14;2021:8863799. doi: 10.1155/2021/8863799 (PMC7822694; doi:10.1155/2021/8863799)

**A**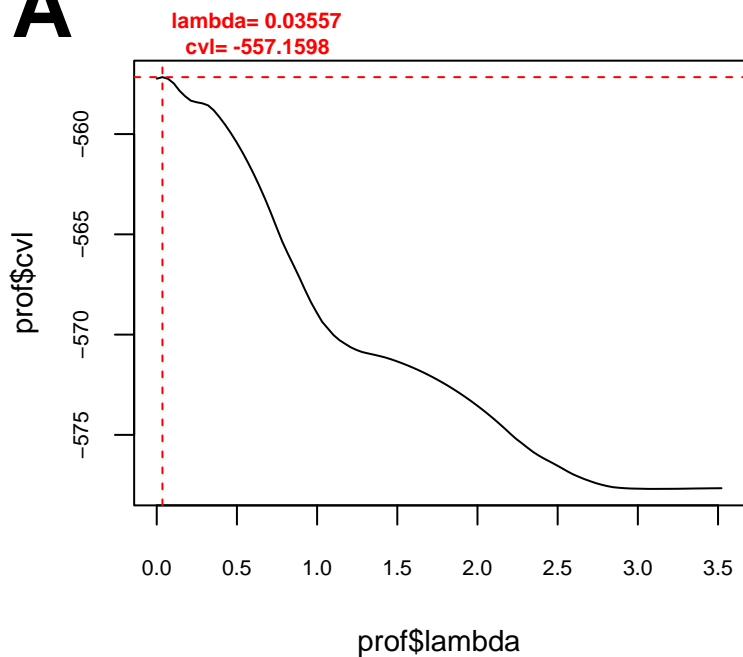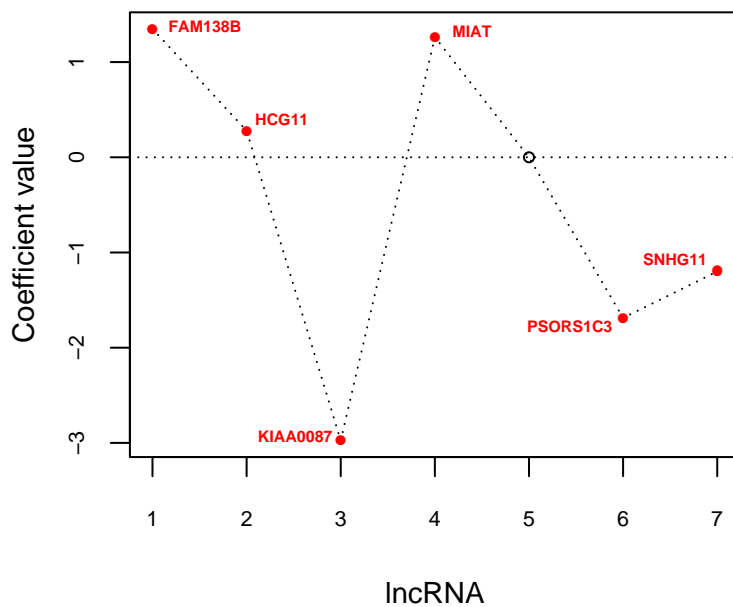**B**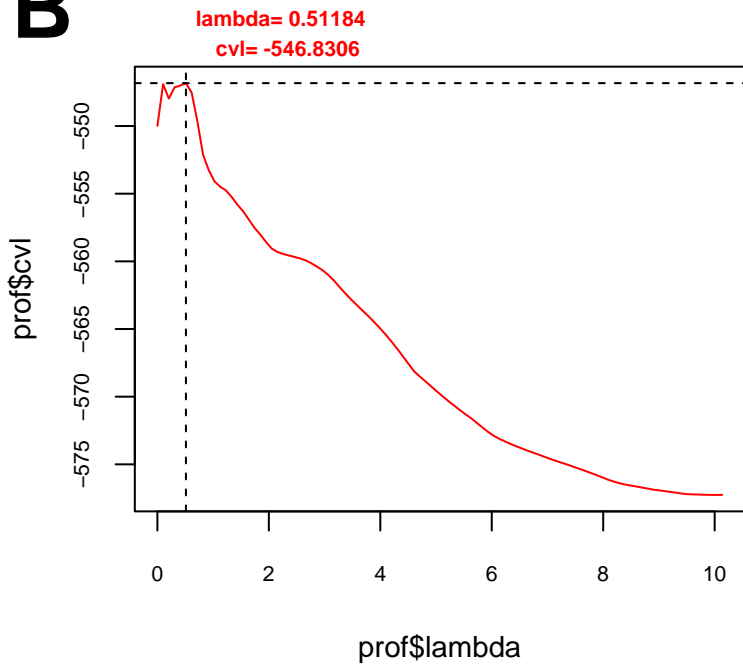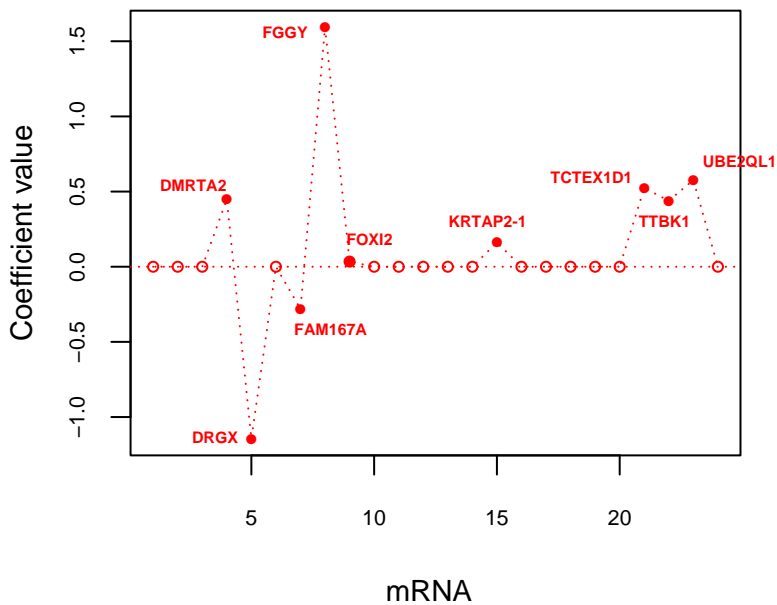

Supplement: Supplementary Materials — Figure S1: the lambda parameter curve (left) and coefficient distribution chart (right) of (a) lncRNA and (b) mRNA. The lambda parameter curve was gained basing on cross-validation likelihood screening. The horizontal axis and vertical axis represent the different values of lambda and CVL, respectively, and the intersection of red dotted lines indicates the value of lambda parameters when CVL reaches the maximum value. The coefficient distribution charts for optimal prognosis were screened basing on Cox-PH model using L1-penalized regularized regression algorithm. [file 8863799.f1.pdf]
